# Consistencies and differences in intermediate physiological phenotypes of vascular aging between ischaemic stroke aetiologies

**DOI:** 10.1159/000525764

**Published:** 2022-08-23

**Authors:** Alastair John Stewart Webb, Karolina A Wartolowska, Linxin Li, Sara Mazzucco, Peter M Rothwell

**Affiliations:** * Wolfson Centre for Prevention of Stroke and Dementia, University of Oxford, UK

**Keywords:** Hypertension, arterial stiffness, blood pressure variability, stroke

## Abstract

**Objective:**

Arterial stiffness, cerebral pulsatility and beat-to-beat blood pressure variability partly mediate the relationship between hypertension and stroke but it is unknown if these intermediate phenotypes of vascular aging differ between stroke aetiologies. We therefore aimed to characterise differences in these intermediate cardiovascular phenotypes between patients presenting with strokes of different aetiologies.

**Methods:**

In consecutive patients on best medical management one month after TIA or non-disabling stroke (Oxford Vascular Study), arterial stiffness (PWV) was measured by applanation tonometry (Sphygmocor), middle cerebral blood flow velocity and pulsatility index (MCA-PI) were measured by transcranial ultrasound (TCD, DWL DopplerBox) and beat-to-beat BP variability was measured with a Finometer. Differences between patients with large artery (LAS), small vessel (SVD), cardioembolic (CE) or undetermined events were derived, including adjustment for cardiovascular risk factors. Relationships were characterised by mixed linear models.

**Results:**

In 909 eligible patients, MCA-PI, PWV and SBPV were all positively skewed. Mean values were greatest in LAS than CE and lowest in SVD (p<0.001). However, after adjustment for age, sex and risk factors, PI was greatest in LAS and lowest in CE stroke, whilst PWV was greatest in SVD and undetermined stroke (p<0.001). In multivariate linear models, age was more strongly associated with PWV and PI in patients with small vessel stroke than other aetiologies, particularly under the age of 65, but SBPV was only weakly associated with demographic indices in all stroke subtypes.

**Conclusions:**

Intermediate cardiovascular phenotypes of vascular aging had similar demographic associations between stroke aetiologies but these were particularly strong in patients with small vessel stroke under the age of 65, implying a potential role of these phenotypes in increasing stroke risk in this patient group.

## Introduction

Rapid assessment and initiation of secondary prevention after transient ischaemic attack or minor stroke reduces early recurrent ischaemic stroke by up to 80% [1], whilst initiation of effective antihypertensive treatment[2], dual antiplatelet therapy[3, 4], intensive lipid lowering[5], anticoagulation for atrial fibrillation[6] and interventions for carotid stenosis[7] and patent foramen ovale[8, 9] reduce the long-term risk of recurrent stroke. However there remains a greater than 10% residual 5-year risk of recurrent ischaemic stroke and major adverse cardiovascular events[10] that is not adequately explained by poor compliance or resistance to current treatments[11].

Hypertension is the underlying cause of up to 60% of strokes. In large artery disease and cardioembolic strokes, hypertension induces stroke through intermediate cardiovascular phenotypes such as carotid stenosis[7] or atrial fibrillation, but no specific intermediate cardiovascular phenotype exists for small vessel disease (SVD). Having identified blood pressure variability as a novel risk factor for recurrent stroke,[12, 13] we incepted a cohort to identify physiological determinants of blood pressure variability, demonstrating strong relationships between blood pressure variability, arterial stiffness and arterial pulsatility[14], their progression over time[15] and their prognostic significance,[16] particularly in SVD.[17] These intermediate phenotypes between hypertension and age with cerebrovascular events are together key manifestations of vascular aging, but the relative importance of these phenotypes in different stroke aetiologies is unclear.

We therefore determined differences in arterial stiffness, cerebral blood flow and blood pressure variability between patients with cerebrovascular events of each aetiology, and, within each subtype, their relationship with age and cardiovascular risk factors as a measure of their role as intermediate phenotypes of vascular aging.

## Methods

### Study population

Consecutive, consenting patients with TIA or minor stroke were recruited from 20102019 to the Phenotyped Cohort of the Oxford Vascular Study (OXVASC)[18]. Participants were recruited at the OXVASC daily emergency clinic, following attendance at the Emergency Department or referral from primary care, usually within 24 hours. The OXVASC population consists of >92,000 individuals registered with about 100 primary-care physicians in Oxfordshire, UK. All consenting patients underwent a standardised medical history and examination, ECG, blood tests and a stroke protocol MRI brain and contrast-enhanced MRA (or CT-brain and carotid Doppler ultrasound or CT-angiogram), an echocardiogram and 5 day ambulatory cardiac monitor. All patients were assessed by a study physician, reviewed by the senior study neurologist (PMR) and followed-up face-to-face up to 10 years. Definition of stroke aetiology was carried out by TOAST classification by a panel of stroke physicians and neurologists chaired by the senior study neurologist since study inception, after completion of all routine investigations. Strokes or TIAs with ‘other aetiology’ in the TOAST classification, multiple possible aetiologies, or incomplete investigation were excluded from this analysis.

As part of the OXVASC Phenotyped cohort, a routine prospective cardiovascular physiological assessment is performed at 1 month follow-up visit.[14] Participants were excluded from specific tests if under 18 years, cognitively impaired (MMSE<23), pregnant, had autonomic failure, a recent myocardial infarction, unstable angina, heart failure (NYHA 3-4 or known ejection fraction <40%) or untreated bilateral carotid stenosis (>70%).

Physiological tests were performed at rest in a quiet, dimly-lit, temperature-controlled room (21-23^0^C). Continuous ECG and non-invasive blood pressure were acquired at 200Hz (Finometer, FMS), via a Powerlab 8/30 with LabChart Pro software (ADInstruments, USA). Automated calibration was performed until the recording was stable, but turned off during testing. Estimated brachial waveforms (Finometer) were calibrated offline by linear regression to 2-3 supine, oscillometric brachial readings, performed immediately prior to the monitoring period on the contralateral arm, with manual exclusion of artefacts. In patients with a significant deterioration in recording quality during the first five minutes, the test was stopped, and the calibration procedure repeated. If necessary the cuff was moved to an adjacent finger or the proximal phalanx of the same finger, or the hand was warmed with a hand warmer. Prior to physiological assessment, two sitting clinic BPs, 5 minutes apart, were measured at ascertainment and one month in the non-dominant arm, by trained personnel.

Blood pressure variability (BPV) on beat-to-beat monitoring was calculated over 5 minutes. Ectopic beats and artefacts were automatically detected from the R-R interval of the ECG, visually reviewed and removed by linear interpolation of R-R interval. Blood pressure artefacts were automatically detected and manually reviewed, and removed by linear interpolation to adjacent normal beats, with in-house software. Systolic and diastolic BPV were calculated as the coefficient of variation (CV=SD/mean), after de-trending of the recording about a linear regression across 5 minutes. All recordings were reviewed blinded to clinical data, after automated and manual data cleaning, to assess for the quality of recording (1 - excellent quality; 2- adequate quality for analysis; 3-unuseable, poor quality recording), based upon the presence of artefacts or drift in the baseline measurement.

Transcranial ultrasound (Doppler Box, Compumedics DWL, Singen, Germany) was performed with a 2MHz probe at the temporal bone window on the same side as carotid applanation, where possible. The MCA was insonated at the site of peak velocity closest to 50mm, or if this was not adequate, at the depth giving the optimal waveform, excluding vessels with velocity transitions and magnitude indicative of a focal MCA stenosis. All waveforms were visually inspected and beats corrupted by artefact were excluded. Peak (PSV), end-diastolic (EDV) and mean (MV) velocities were calculated as the average of the remaining beats during a 15 second window, from the envelope of the spectrum. MCA pulsatility was calculated as Gosling’s pulsatility index (MCA-PI= (systolic CBFV-diastolic CBFV) / mean CBFV).

Applanation tonometry (Sphygmocor, AtCor Medical, Sydney, Australia) was used to measure carotid-femoral pulse wave velocity (aortic-PWV), aortic augmentation index and aortic systolic and diastolic blood pressure and pulse pressure (ao-SBP, ao-DBP, ao-PP). Other indices measured in this cohort[14, 19] (cerebrovascular reactivity, autoregulation, reactivity to stress) are not intermediate markers of vascular aging, with no demonstrated association with aging, and their importance as mediators of clinical outcomes is unproven. Further research is required to develop analysis of these measures prior to understanding any differences between stroke aetiologies..

Differences in demographic indices were compared between aetiological groups by chi-squared, ANOVA and t-tests. Distributions for each index were stratified by aetiological subtype and sex, and plotted as kernel density plots.

The key outcome of the difference in the three primary variables (PWV, PI and SBPV) between aetiological subtypes was determined by ANCOVA, before and after adjustment for age, sex, hypertension, diabetes, current smoking and BMI, presented as estimated marginal means. Associations between each of the core variables and cardiovascular risk factors, specifically the relationship with age as an index of their role as intermediate phenotypes of vascular, were determined by general linear models. Analyses were repeated for major physiological determinants of the core indices (mean systolic and diastolic blood pressure and cerebral blood flow velocities). A p-value <0.05 is taken to indicate statistical significance.

Analyses were performed in R and Matlab r2018, using the packages *tidyverse, lme4, emeans* and in-house software. The data that support the findings of this study are available from Prof Rothwell (peter.rothwell@ndcn.ox.ac.uk) upon reasonable request.

## Results

909 of 980 patients with valid recordings had a single specific or undetermined stroke aetiology after investigation. 812 had arterial stiffness assessments at baseline, 845 had beat-to-beat BPV and 704 participants had transcranial ultrasound performed. Patients presenting with stroke due to large artery disease were older (p<0.001) and more likely to be male (p=0.014)or diabetic (p=0.0019) (supplementary table 1), whereas patients with small vessel strokes were more likely to have a history of smoking.

There were similar, positively-skewed distributions for PWV, MCA-PI and beat-to-beat BPV, although this was less marked for cerebral pulsatility than for PWV or SBPV (supplementary figures 1-2). The shape of the distributions was consistent for patients with large vessel, cardioembolic, small vessel and undetermined stroke (Supplementary figure I), but the positive skew was more evident in the elderly (Supplementary figure 2).

In unadjusted associations, physiological indices were similar between stroke aetiologies, although there was a trend to greater PI and PWV in large vessel stroke followed by cardioembolic stroke, with the lowest values in small vessel stroke (table 1). However, after adjustment for age and sex, the estimated marginal means for PWV, PI and SBPV remained similar for patients with large vessel disease or cardioembolic stroke, but PWV and PI increased in patients with SVD to a similar level to large vessel disease (table 1). Adjustment for cardiovascular risk factors resulted in the greatest PI in LAS and lowest in CE stroke, with similar values between SVD and undetermined events, with greater aortic blood pressure measures in SVD after adjustment (supplementary table 2). In contrast, PWV was greatest in SVD and undetermined stroke compared to LAS and CE. Mean values of SBPV were largely unaffected by adjustment for age, sex or cardiovascular risk factors.

There were limited differences between groups in mean values of blood flow velocity measures in both the aorta and the middle cerebral artery (supplementary table 1). Aortic DBP was greater in patients with SVD related stroke, reflecting the younger age of this group, but this attenuated following adjustment for age, sex and cardiovascular risk factors. In adjusted linear regressions, a low aortic DBP, high aortic PP and low end-diastolic velocity (EDV) were all strongly associated with age, with the strongest association in patients with SVD (supplementary table 3).

**Figure 1:**
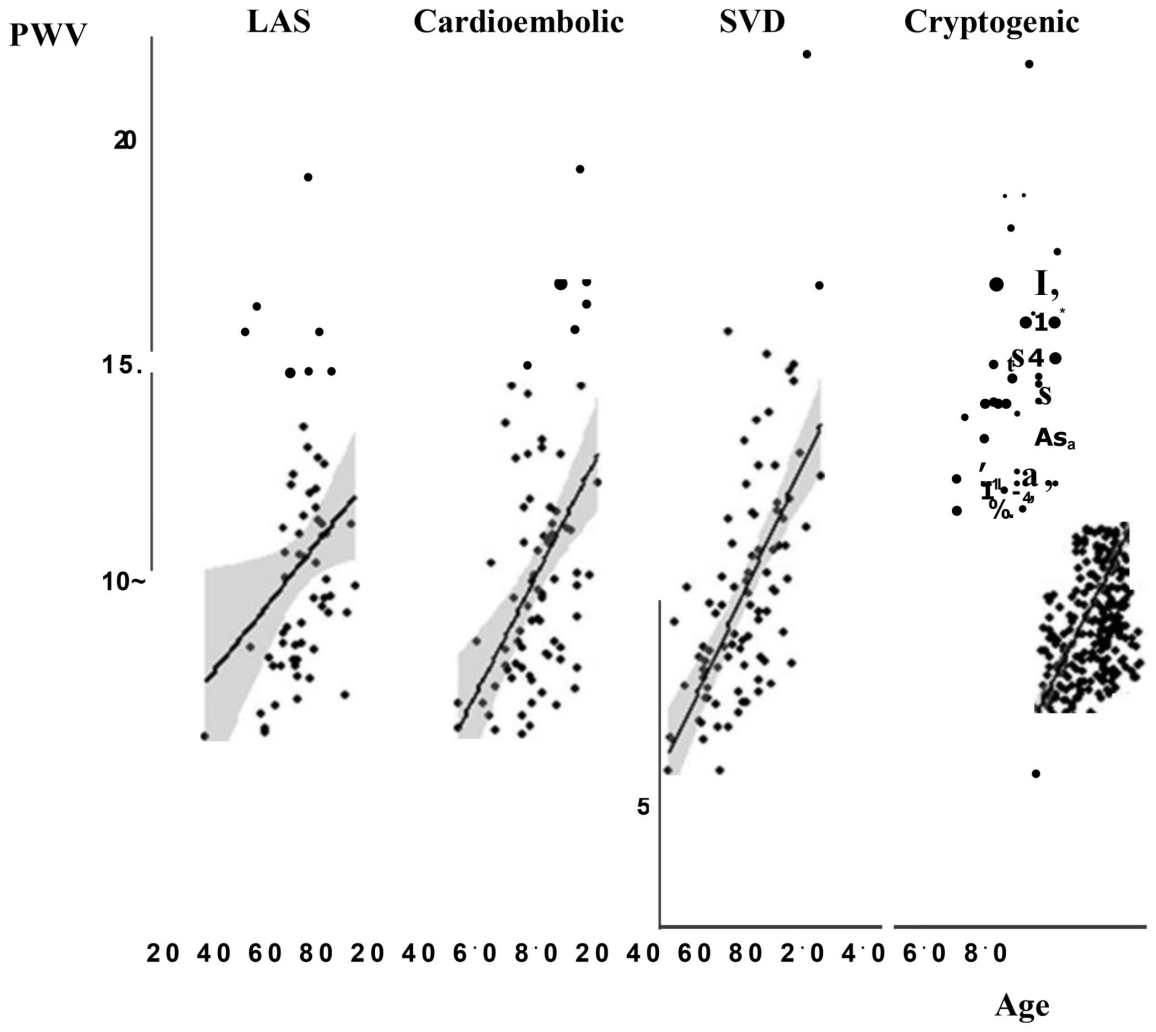
Distribution of arterial stiffness stratified by stroke aetiology. Scatter plots with linear regression lines and 95% confidence intervals.

There were similar directions and magnitudes of associations between cardiovascular phenotypes (PI, PWV, SBPV) and age for patients in each stroke aetiology. However, the strength of association between age and PWV or PI was greater in patients with SVD (figure 1+2, supplementary figures 3-4, table 2) and undetermined stroke, than patients with large vessel or cardioembolic stroke. In contrast to PWV and PI, SBPV was less strongly associated with age with a weak association in patients with cardioembolic or large vessel stroke, but no association in patients with SVD-related stroke. The relationship between age and PI or PWV was best described by a linear model for all aetiologies, including SVD, except for undetermined stroke where a quadratic model allowing for an increasing rate of association with PWV at increasing ages (supplementary table 4). However, there were too few patients with large artery or cardioembolic stroke below to age of 65 to reliably determine if the relationship differed at younger ages although in patients under 65, age was still strongly associated with cerebral pulsatility in patients with SVD (p=0.002), with no significant association in undetermined stroke (p=0.06), LAS or CE stroke (p=0.19, p=0.81 respectively).

## Discussion

There were consistent associations between aortic stiffness, cerebral pulsatility and beat-to-beat blood pressure variability with age and other demographic indices in patients with cardioembolic, large vessel, small vessel and undetermined stroke. However, after adjustment for age and cardiovascular risk factors, cerebral pulsatility was similar in patients with large vessel or small vessel stroke and lower in cardioembolic stroke, but arterial stiffness (PWV) was greatest in small vessel and undetermined stroke, reflecting greater vascular end organ damage at a younger age. Similarly, linear associations between age and either PI or PWV were stronger in patients with SVD than large vessel or cardioembolic stroke aetiologies, particularly under 65.

PWV, PI[20] and beat-to-beat BPV are all associated with age and hypertension in cerebrovascular[16], and general populations[21], and predict recurrent cardiovascular events, independent of age and other risk factors[16]. However, there is minimal evidence for differences between these markers of vascular aging in patients with different stroke aetiologies. The stronger association between PWV with age in patients with SVD, with a significant relationship under the age of 65, implies that although the underlying physiological relationship between age and arterial stiffness is the same, vascular aging occurs earlier and may be more closely linked to the development of small vessel arteriopathy than either atrial fibrillation or carotid stenosis. Previous studies demonstrated a relationship between aortic stiffness and cerebral arterial pulsatility, cerebral white matter hyperintensities[22, 17, 23] and lacunar stroke[24] and visit-to-visit BPV with cerebral SVD[25]. This is consistent with either a causative role of increased arterial stiffness with SVD, reflecting greater transmission of aortic pulsatility to the brain.

Cerebral pulsatility was higher in patients with large vessel disease than cardioembolic stroke, even after adjustment for cardiovascular risk factors, to a similar extent to SVD. This is consistent with blood pressure pulsatility being relevant to stroke due to large vessel disease in older adults demonstrated in Mendelian Randomisation studies[26, 27].

Beat-to-beat SBPV was only weakly associated with age, and only in patients with cardioembolic or undetermined strokes. This may reflect the lower reproducibilty of beat-to-beat BPV[16, 28, 29], or it may reflect independence of beat-to-beat SBPV from age and other cardiovascular risk factors indicative of a novel causative mechanism[16, 25].

This study is the first population based cohort to systematically measure these indices in unselected, high risk patients with detailed phenotyping and a reliable definition of stroke aetiology[18]. However, there are limitations. Firstly, there are a relatively high proportion of patients with cryptogenic events or events with more than one possible aetiology, reflecting the high proportion of patients with TIA. However, this is a real world sample that reflects clinical practice. Secondly, cardioembolic stroke is principally due to atrial fibrillation which can affect the validity of measurement of blood pressure and pulse wave velocity. Thirdly, despite the relatively large size of this study, the total number of patients in each aetiological group remains relatively limited. However, the consistency of associations between groups supports the validity of the results. Fourthly, as the large difference in age between aetiological groups confounded the association with markers of vascular aging, this may reflect differences in the association between stroke aetiology and age causing over-correction of the model rather than revealing a true underlying association. Fifthly, some patients with specific causes of large artery or cardioembolic stroke may have been excluded due to the inclusion criteria for the study, although these numbers would likely be small. Finally, analyses were not adjusted for multiple comparisons, but even if all the significant results were conservatively adjusted for three outcomes by setting the threshold for significance at 0.0167, the majority of significant findings would remain.

The stronger association between PWV and PI with age in patients with SVD, and the increased mean values after adjustment, suggests that SVD-related stroke may be pathophysiologically related to age and hypertension to a greater extent than large artery disease or atrial fibrillation, and reflect premature vascular aging in both the great vessels and the brain. However, further research is required to confirm this association in other patient groups, and to determine the strength of this association in patients with other markers of SVD. Specifically, trials are required to identify interventions that could reduce PWV, PI or SBPV, independently of effects on blood pressure, to reliably determine their potential causative role in development of SVD, and identify novel treatments.

In addition to the pathophysiological implications of these findings, the results imply that clinical approaches that target these intermediate cardiovascular markers could be beneficial in all stroke subtypes, but may be of greater benefit in younger patients with SVD. For example, the results demonstrate a strong association with a history of hypertension for cerebral pulsatility. This supports the need for early, effective blood pressure control, both to reduce the direct risk of stroke associated with hypertension but potentially also to prevent the development of intermediate cardiovascular phenotypes such as increased cerebral pulsatility, which have uncertain reversibility. Finally, the mean values and distribution of these indices (table 1, supplementary figures) in this population and provide an aetiology-specific reference range for clinical practice.

Overall, age was similarly associated with arterial stiffness, cerebral arterial pulsatility and beat-to-beat blood pressure variability in patients with large artery, small vessel or cardioembolic stroke. However, mean values of PWV and PI were greater in patients with large artery disease than cardioembolic stroke and in patients with small vessel stroke than other forms of stroke, after adjustment for age. Similarly, there was a much stronger relationship between age and PWV or PI in patients with small vessel stroke than in patients with cardioembolic or large artery stroke.

## Supplementary Material

Supplemental

## Figures and Tables

**Figure 2:**
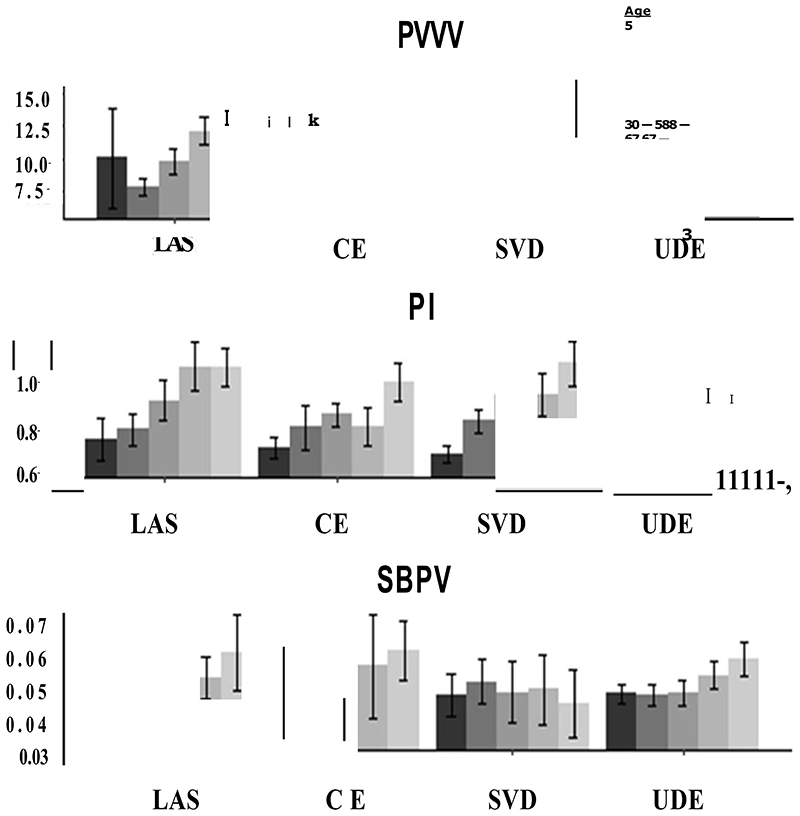
Distribution of arterial stiffness (pulse wave velocity), pulsatility index and beat-to-beat BP variability, stratified by stroke aetiology and quintiles of age

**Table 1:** Differences between aetiological stroke subtypes and each intermediate cardiovascular phenotype. Results are shown as estimated marginal means for general linear models unadjusted, adjusted for age and sex and adjusted for age, sex and cardiovascular risk factor. p-values are shown for ANCOVA with the same covariates. PI = pulsatility index; PWV = pulse wave velocity; SBPV = systolic blood pressure variability (residual coefficient of variation); RFs= risk factors, including history of hypertension, diabetes, current smoking and BMI; SVD = small vessel disease; CI = confidence interval.

	Large Artery		Cardioembolic		SVD		Undetermined		p	
	Value	95%CI	Value	95%CI	Value	95%CI	Value	95%CI	p-value
**Unadjusted**									
PI	0.90	(0.85 - 0.94)	0.80	(0.76 - 0.85)	0.76	(0.72 - 0.8)	0.80	(0.78 - 0.82)	0.007
PWV (m/s)	10.4	(9.68 - 11.1)	10.1	(9.5 - 10.7)	9.69	(9.1 - 10.3)	9.57	(9.3 - 9.84)	0.014
SBPV	0.049	(0.045 - 0.054)	0.05	(0.046 - 0.054)	0.045	(0.041 - 0.049)	0.047	(0.045 - 0.049)	0.16
**Adjusted Age/Sex**									
PI	0.86	(0.82 - 0.9)	0.77	(0.73 - 0.8)	0.81	(0.78 - 0.85)	0.81	(0.79 - 0.82)	0.34
PWV (m/s)	9.68	(9.08 - 10.3)	9.4	(8.87 - 9.92)	10.2	(9.73 - 10.7)	9.67	(9.44 - 9.89)	0.93
SBPV	0.049	(0.044 - 0.053)	0.049	(0.045 - 0.054)	0.046	(0.042 - 0.05)	0.047	(0.045 - 0.049)	0.32
**Adjusted Age/Sex/RFs**									
PI	0.88	(0.84 - 0.93)	0.80	(0.76 - 0.84)	0.84	(0.8 - 0.88)	0.84	(0.81 - 0.86)	<0.0001
PWV (m/s)	9.72	(9.1 - 10.3)	9.85	(9.26 - 10.4)	10.7	(10.1 - 11.2)	10.3	(9.99 - 10.7)	<0.0001
SBPV	0.053	(0.048 - 0.058)	0.052	(0.047 - 0.057)	0.049	(0.045 - 0.053)	0.05	(0.047 - 0.053)	0.15

**Table 2:** Associations between demographic and vascular indices, stratified by stroke aetiology. Results are shown as beta coefficients and p-values for general linear models, adjusted for all presented indices. PI = pulsatility index; PWV = pulse wave velocity; SBPV = systolic blood pressure variability (residual coefficient of variation); RFs= risk factors, including history of hypertension, diabetes, current smoking and BMI; SVD = small vessel disease; CI = confidence interval.

	Large Artery		Cardioembolic		SVD		Undetermined	
	Beta	p-val	Beta	p-val	Beta	p-val	Beta	p-val
PWV (m/s)								
Age (years)	0.8	0.012	1.3	0.00013	1.5	<0.0001	1.5	<0.0001
Female	0.012	0.97	0.065	0.83	-0.14	0.55	-0.14	0.55
Hypertension	0.094	0.77	0.65	0.037	0.34	0.13	0.34	0.13
Diabetes	1.3	0.00013	0.41	0.18	-0.036	0.88	-0.036	0.88
Smoking	0.23	0.44	0.17	0.58	-0.26	0.27	-0.26	0.27
BMI (Kg/m^2^)	0.041	0.9	0.28	0.38	-0.043	0.86	-0.043	0.86
PI								
Age (years)	0.08	0.014	0.074	0.003	0.13	<0.0001	0.1	<0.0001
Female	0.0098	0.73	0.059	0.007	0.023	0.096	0.01	0.15
Hypertension	0.061	0.049	0.04	0.085	0.0012	0.93	0.028	0.00023
Diabetes	0.03	0.34	-0.019	0.36	0.0053	0.73	0.022	0.0022
Smoking	-0.009	0.76	0.022	0.32	0.01	0.5	0.003	0.67
BMI (Kg/m^2^)	-0.055	0.083	0.0032	0.88	0.006	0.71	-0.011	0.15
SBPV								
Age (years)	0.0035	0.18	0.007	0.023	0.0022	0.31	0.004	<0.0001
Female	0.001	0.66	0.0039	0.16	0.00004	0.83	0.0012	0.2
Hypertension	0.0015	0.56	-0.0051	0.086	-0.00044	0.82	-0.0012	0.23
Diabetes	-0.0015	0.56	0.0028	0.32	0.0023	0.26	0.00047	0.61
Smoking	0.0033	0.18	0.0043	0.14	0.0045	0.027	0.0013	0.15
BMI (Kg/m^2^)	0.0042	0.12	0.0033	0.25	0.0022	0.32	0.001	0.28
