# Supplementary material for: Consistencies and differences in intermediate physiological phenotypes of vascular aging between ischaemic stroke aetiologies
Source: Cerebrovasc Dis. Author manuscript; Available in PMC 2024 May 27. (PMC7615998; doi:10.1159/000525764)
Supplement: Supplemental [file EMS186815-supplement-Supplemental.docx]

SUPPLEMENTARY DATA

Consistencies and differences in intermediate physiological phenotypes between ischaemic stroke aetiologies

Webb AJS, Lawson A, Wartolowska K, Li L, Mazzucco S, Rothwell PM

**Supplementary Table 1. Demographic characteristics of patients stratified by aetiological subtypes.** Values reported are number and percentage for discrete measures, and mean and standard deviation for continuous indices.

|  | **Large Artery** | **Cardioembolic** | **SVD** | **Undetermined** | **p** |
| --- | --- | --- | --- | --- | --- |
| n | 96 | 109 | 113 | 591 |  |
| Age (years) | 72.2 (10) | 72.3 (12) | 62 (13) | 65.8 (13) | <0.0001 |
| Female | 30 (31) | 47 (43) | 51 (45) | 288 (49) | 0.014 |
| Event  TIA | 57 (59) | 61 (56) | 51 (45) | 426 (72) | <0.0001 |
| Stroke | 39 (41) | 48 (44) | 62 (55) | 165 (28) |  |
| Hypertension | 83 (86) | 98 (90) | 87 (77) | 435 (74) | 0.0003 |
| Diabetes | 22 (23) | 13 (12) | 17 (15) | 57 (9.6) | 0.0019 |
| Atrial fibrillation | 1 (1) | 95 (87) | 0 (0) | 7 (1.2) | <0.0001 |
| Dyslipidaemia | 51 (53) | 61 (56) | 78 (69) | 376 (64) | 0.048 |
| Smoker Ever | 61 (65) | 59 (54) | 69 (61) | 292 (49) | 0.010 |
| Smoker Current | 14 (15) | 11 (10) | 36 (32) | 90 (15) | <0.0001 |
| Medications: |  |  |  |  |  |
| Antiplatelet | 90 (94) | 63 (58) | 101 (89) | 527 (89) | <0.0001 |
| Antihypertensives | 80 (83) | 95 (87) | 83 (73) | 427 (72) | 0.0023 |
| Statins | 90 (94) | 82 (75) | 93 (82) | 450 (76) | 0.0008 |
| Weight (Kg) | 78.4 (15) | 81.6 (19) | 80.5 (19) | 78.2 (18) | 0.28 |
| BMI (Kg/m2) | 27.4 (4.4) | 27.7 (5) | 28 (6) | 27.1 (5.1) | 0.23 |
| Creatinine | 89.5 (24) | 82.7 (28) | 76.8 (21) | 75 (19) | <0.0001 |
| SBP (mmHg) | 136 (21) | 135 (21) | 133 (19) | 133 (19) | 0.1 |
| DBP (mmHg) | 74.9 (13) | 79.2 (13) | 79.6 (12) | 77.7 (11) | 0.45 |

**Supplementary Figure 1.** **Relationship between age and pulsatility index (PI) stratified by stroke aetiology.** Scatter plots stratified by gender (men=red; women=blue), with linear regression lines and 95% confidence intervals. LAS – large artery stroke; SVD = small vessel disease; PI = pulsatility index

**Age (years)**

**PI**

**LAS Cardioembolic SVD Cryptogenic**


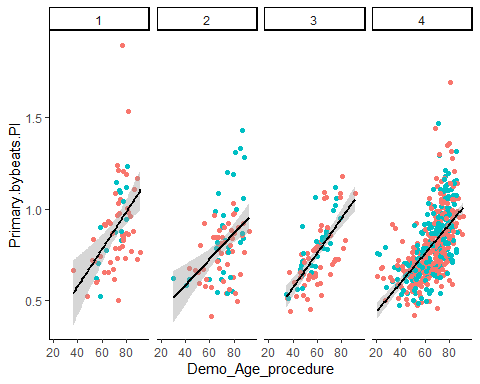


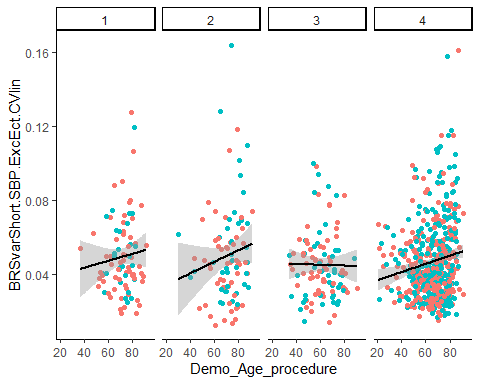
**Supplementary Figure 2. Relationship between age and beat-to-beat blood pressure variability (BPV) stratified by stroke aetiology.** Scatter plots stratified by gender (men=red; women=blue), with linear regression lines and 95% confidence intervals. LAS – large artery stroke; SVD = small vessel disease; BPV = blood pressure variability.

**Age (years)**

**BPV**

**LAS Cardioembolic SVD Cryptogenic**

**Age**

**PI**

**LAS Cardioembolic SVD Cryptogenic**

**Supplementary Figure 3.** Distribution of arterial stiffness (pulse wave velocity), pulsatility index and beat-to-beat BP variability, stratified by stroke aetiology. PWV = pulse wave velocity

**Large Artery**

**Cardioembolic**

**Small Vessel**

**Undefined**


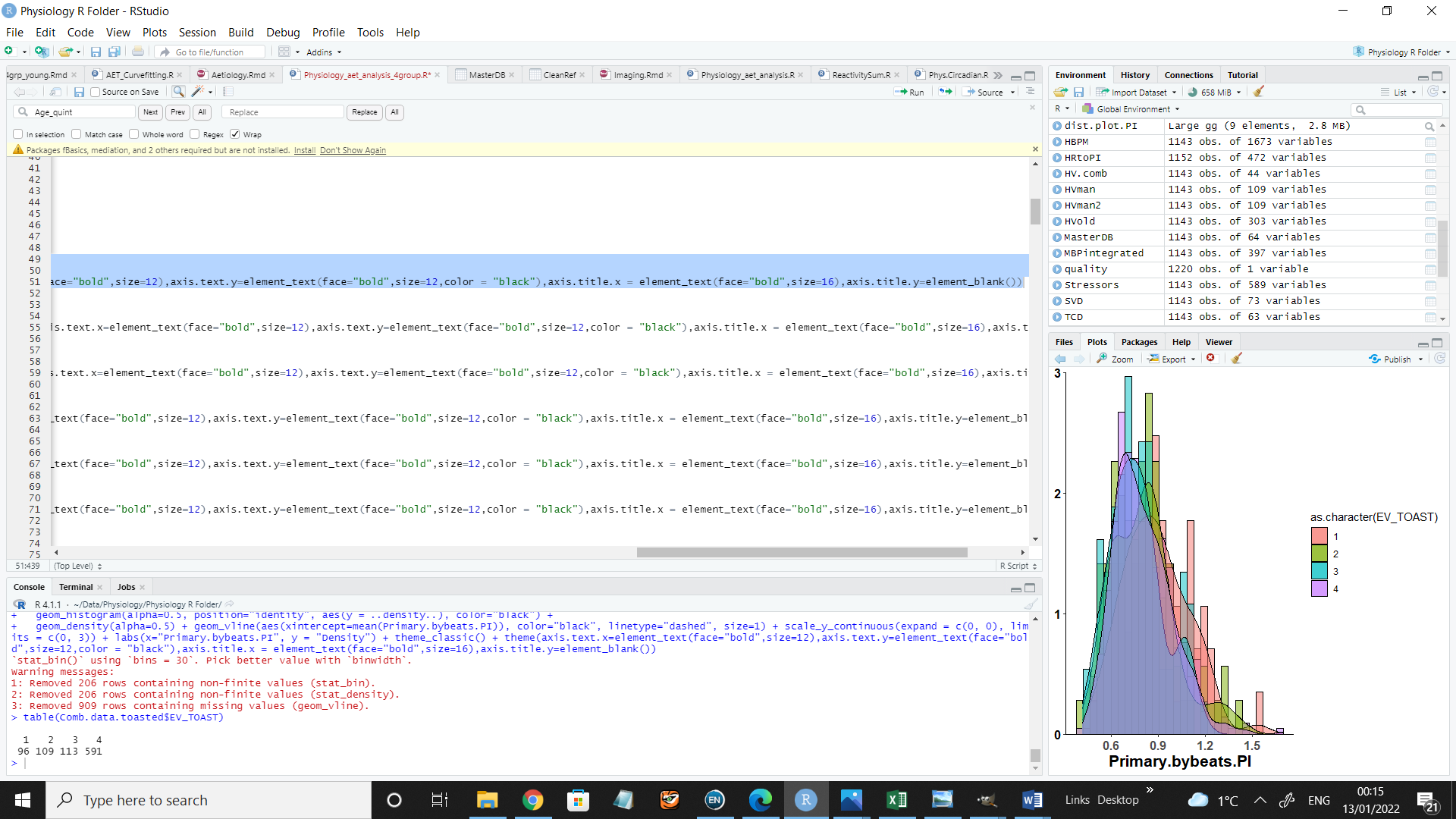

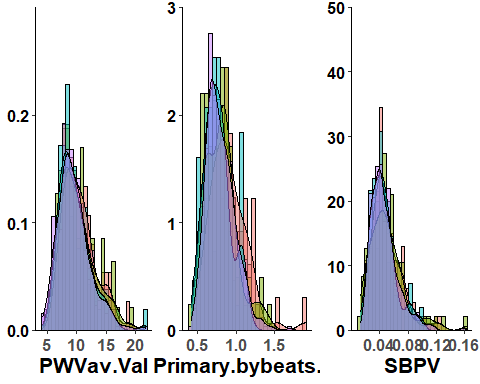


**PWV (m/s) Pulsatility Index Systolic BPV**

**Supplementary Figure 4.** Distribution of arterial stiffness (pulse wave velocity), pulsatility index and beat-to-beat BP variability, stratified by stroke aetiology and age above or below 60 years

**Large Artery**

**Cardioembolic**

**Small Vessel**

**Undefined**


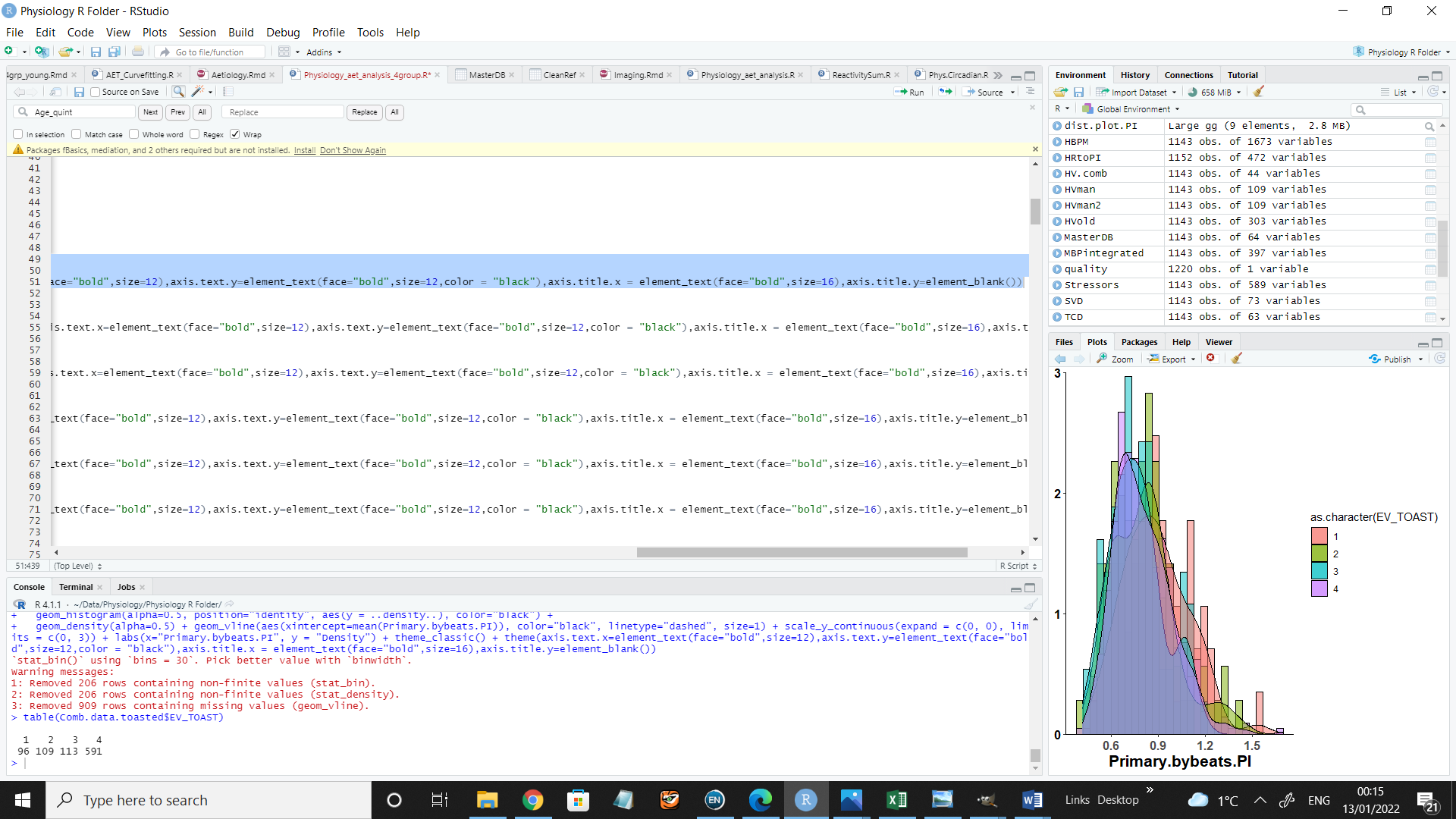

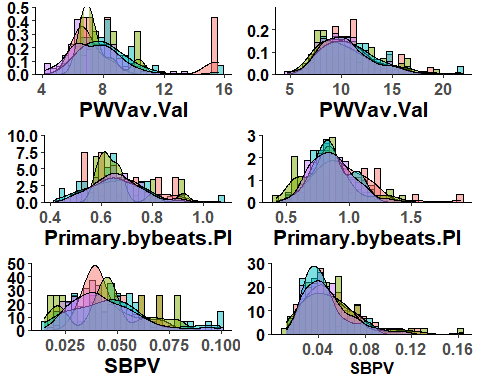


**Pulse Wave Velocity**

**Pulse Wave Velocity**

**Pulsatility Index**

**Pulsatility Index**

**Systolic BPV**

**Systolic BPV**

**Age <60**

**Age >60**

**Supplementary Table 2. Differences between aetiological stroke subtypes and blood flow indices.** Results are shown as estimated marginal means for general linear models unadjusted, adjusted for age and sex and adjusted for age, sex and cardiovascular risk factor. p-values are shown for ANCOVA with the same covariates. Blood pressure indices are given in mmHg and ultrasound indices in cm/s. SBP = Systolic blood pressure; DBP = diastolic blood pressure; PP = pulse pressure; PSV= peak systolic velocity; EDV = end diastolic velocity; SVD = small vessel disease; CI = confidence interval.

| . | **Large Artery** | | **Cardioembolic** | | **SVD** | | **Undetermined** | | **p** |
| --- | --- | --- | --- | --- | --- | --- | --- | --- | --- |
|  | Value | 95%CI | Value | 95%CI | Value | 95%CI | p-value | 95%CI | p-value |
| **Unadjusted** |  |  |  |  |  |  |  |  |  |
| Aortic SBP | 124 | (120 - 127) | 122 | (119 - 126) | 122 | (118 - 125) | 121 | (119 - 122) | 0.12 |
| Aortic DBP | 72.5 | (70.5 - 74.5) | 73.4 | (71.6 - 75.3) | 76.2 | (74.4 - 78.1) | 73.1 | (72.3 - 74) | 0.96 |
| Aortic PP | 54 | (50.6 - 57.3) | 49.9 | (46.7 - 53.1) | 49.8 | (46.7 - 52.9) | 50.2 | (48.9 - 51.6) | 0.14 |
| PSV | 86 | (82 - 91) | 76 | (72 - 81) | 81 | (77 - 85) | 82 | (80 - 83) | 0.91 |
| EDV | 39 | (37 - 42) | 37 | (34 - 39) | 41 | (38 - 43) | 40 | (39 - 41) | 0.18 |
|  |  |  |  |  |  |  |  |  |  |
| **Adjusted** |  |  |  |  |  |  |  |  |  |
| Aortic SBP | 121 | (117 - 125) | 120 | (116 - 124) | 125 | (121 - 129) | 122 | (119 - 124) | 1e-07 |
| Aortic DBP | 71 | (68 - 73) | 72 | (70 - 75) | 75 | (73 - 77) | 72 | (71 - 74) | 7.1e-06 |
| Aortic PP | 54 | (50 - 57) | 49 | (46 - 53) | 53 | (50 - 56) | 52 | (50 - 54) | 0.007 |
| PSV | 90 | (85 - 95) | 80 | (75 - 85) | 83 | (78 - 87) | 83 | (80 - 86) | 0.92 |
| EDV | 41 | (39 - 44) | 39 | (36 - 41) | 39 | (37 - 41) | 39 | (38 - 41) | 0.081 |
|  |  |  |  |  |  |  |  |  |  |

**Supplementary Table 3. Associations between demographic indices and blood flow indices, stratified by stroke aetiology.** Results are shown as beta coefficients and p-values for general linear models, adjusted for presented indices. Blood pressure is reported in mmHg and cerebral blood flow in cm/s. SBP = systolic blood pressure; DBP = diastolic blood pressure; PP = pulse pressure; PSV = peak systolic velocity; EDV = end-diastolic velocity.

| . | **Large Artery** | | **Cardioembolic** | | **Small Vessel Disease** | | **Undetermined** | |
| --- | --- | --- | --- | --- | --- | --- | --- | --- |
|  | Beta | p-val | Beta | p-val | Beta | p-val | Beta | p-val |
| **Aortic SBP** |  |  |  |  |  |  |  |  |
| Age (years) | -0.29 | 0.89 | 5.1 | 0.015 | 1.8 | 0.37 | 5.7 | <0.0001 |
| Female | 1.6 | 0.44 | 2.4 | 0.22 | 0.46 | 0.81 | 1.8 | 0.013 |
| Hypertension | 0.18 | 0.93 | 4.5 | 0.026 | 3.5 | 0.072 | 3.9 | <0.0001 |
| Diabetes | 4.8 | 0.026 | -1.2 | 0.54 | -3.2 | 0.12 | -0.68 | 0.36 |
| Smoking | -0.48 | 0.82 | 1.3 | 0.5 | 1.3 | 0.52 | 1.4 | 0.061 |
| BMI (Kg/m^2^) | -4.1 | 0.072 | -1.9 | 0.35 | 0.36 | 0.86 | -1.4 | 0.061 |
|  |  |  |  |  |  |  |  |  |
| **Aortic DBP** |  |  |  |  |  |  |  |  |
| Age (years) | -2.5 | 0.018 | -2.1 | 0.045 | -4.2 | 0.00016 | -1 | 0.023 |
| Female | -1.2 | 0.21 | -1.6 | 0.12 | -1.9 | 0.065 | -1.6 | <0.0001 |
| Hypertension | -1.8 | 0.083 | 2.7 | 0.0092 | 2 | 0.06 | 1.8 | <0.0001 |
| Diabetes | 2.1 | 0.044 | -0.38 | 0.71 | -1.5 | 0.16 | -1.1 | 0.01 |
| Smoking | -0.41 | 0.68 | 0.16 | 0.88 | -0.25 | 0.81 | 0.31 | 0.46 |
| BMI (Kg/m^2^) | 0.045 | 0.97 | -0.66 | 0.53 | 1.1 | 0.31 | -0.41 | 0.32 |
|  |  |  |  |  |  |  |  |  |
| **Aortic PP** |  |  |  |  |  |  |  |  |
| Age (years) | 2.5 | 0.21 | 4.5 | 0.019 | 4.9 | 0.0062 | 6.8 | <0.0001 |
| Female | 1.5 | 0.42 | 5.1 | 0.0029 | 3.2 | 0.047 | 3.4 | <0.0001 |
| Hypertension | 1.3 | 0.5 | 2.3 | 0.19 | 2.7 | 0.11 | 1.1 | 0.081 |
| Diabetes | 2.2 | 0.25 | -0.58 | 0.75 | 1.2 | 0.49 | 0.44 | 0.47 |
| Smoking | -0.59 | 0.75 | 1.4 | 0.45 | 2.2 | 0.18 | 0.83 | 0.18 |
| BMI (Kg/m^2^) | -2.9 | 0.15 | -2 | 0.25 | -2.6 | 0.14 | -0.15 | 0.81 |
|  |  |  |  |  |  |  |  |  |
| **PSV** |  |  |  |  |  |  |  |  |
| Age (years) | -3.3 | 0.36 | -0.17 | 0.94 | -3 | 0.22 | -2.3 | 0.02 |
| Female | 8.1 | 0.019 | 5.2 | 0.0087 | 1.9 | 0.38 | 4 | <0.0001 |
| Hypertension | 0.7 | 0.84 | -1.3 | 0.55 | 1.7 | 0.43 | -0.5 | 0.6 |
| Diabetes | 0.94 | 0.8 | 2.5 | 0.2 | -1 | 0.66 | 0.83 | 0.35 |
| Smoking | 3.6 | 0.3 | 0.13 | 0.95 | -1.8 | 0.44 | 0.6 | 0.51 |
| BMI (Kg/m^2^) | -5.1 | 0.16 | 0.69 | 0.73 | 1.2 | 0.63 | -1.1 | 0.24 |
|  |  |  |  |  |  |  |  |  |
| **EDV** |  |  |  |  |  |  |  |  |
| Age (years) | -4.1 | 0.012 | -2.2 | 0.054 | -6.1 | <0.0001 | -4.6 | <0.0001 |
| Female | 2.3 | 0.13 | 0.57 | 0.57 | 0.29 | 0.78 | 1.6 | 0.00023 |
| Hypertension | -1.1 | 0.47 | -2.1 | 0.059 | 0.62 | 0.57 | -0.77 | 0.098 |
| Diabetes | 0.38 | 0.81 | 2.2 | 0.026 | -0.86 | 0.46 | -0.35 | 0.43 |
| Smoking | 1.7 | 0.25 | -0.54 | 0.61 | -1.5 | 0.2 | 0.058 | 0.9 |
| BMI (Kg/m^2^) | -0.86 | 0.59 | 0.22 | 0.83 | 0.61 | 0.62 | -0.26 | 0.56 |

**Supplementary Table 4. Comparison between linear versus non-linear models for the relationship between age and markers of physiological phenotypes, stratified by stroke aetiology.** Results are shown as r-squared values and AIC for linear, quadratic, exponential and power curves, including age and gender as predictors. A lower AIC indicates a better fitting model. This applies to comparisons between different types of models for the same index and same subgroup, not for comparing between aetiological subtypes. LAS= large artery stroke; CE = cardioembolic; SVD= small vessel disease; UDE=undefined; AIC =Akaike information criterion; PI= pulsatility index; PWV=pulse wave velocity; SBPV = systolic blood pressure variability.

|  | **Aetiology** | **Linear** | | **Polynomial** | | **Exponential** | | **Power** | |
| --- | --- | --- | --- | --- | --- | --- | --- | --- | --- |
|  |  | r^2^ | AIC | r^2^ | AIC | r^2^ | AIC | r^2^ | AIC |
| PI | LAS | 0.2 | -9.1 | 0.19 | -7.54 | 0.25 | -6.68 | 0.25 | -6.69 |
|  | CE | 0.22 | -42.9 | 0.21 | -41.4 | 0.19 | -9.4 | 0.18 | -8.62 |
|  | SVD | 0.49 | -112 | 0.49 | -110 | 0.51 | -68.6 | 0.51 | -68.8 |
|  | UDE | 0.35 | -453 | 0.39 | -480 | 0.38 | -289 | 0.32 | -248 |
| PWV | LAS | 0.043 | 303 | 0.051 | 303 | 0.086 | 4.5 | 0.097 | 3.72 |
|  | CE | 0.19 | 376 | 0.18 | 378 | 0.21 | 4.22 | 0.2 | 4.52 |
|  | SVD | 0.39 | 387 | 0.4 | 386 | 0.43 | -28.2 | 0.41 | -25.2 |
|  | UDE | 0.35 | 1900 | 0.36 | 1890 | 0.4 | -69.7 | 0.37 | -46.5 |
| SBPV | LAS | -0.012 | -430 | -0.023 | -428 | -0.016 | 95.5 | -0.016 | 95.6 |
|  | CE | 0.03 | -402 | 0.027 | -401 | 0.031 | 137 | 0.027 | 137 |
|  | SVD | -0.019 | -542 | -0.01 | -542 | -0.018 | 113 | -0.018 | 113 |
|  | UDE | 0.021 | -2790 | 0.042 | -2800 | 0.01 | 554 | 0.0049 | 557 |
|  |  |  |  |  |  |  |  |  |  |
